# Gender and sexuality: emerging perspectives from the heterosexual epidemic in South Africa and implications for HIV risk and prevention

**DOI:** 10.1186/1758-2652-13-6

**Published:** 2010-02-09

**Authors:** Rachel Jewkes, Robert Morrell

**Affiliations:** 1 Gender & Health Research Unit, Medical Research Council, Private Bag X3985, Pretoria 0001 South Africa; 2 Research Office, University of Cape Town, P/Bag, Rondebosch 7701, South Africa

## Abstract

Research shows that gender power inequity in relationships and intimate partner violence places women at enhanced risk of HIV infection. Men who have been violent towards their partners are more likely to have HIV. Men's behaviours show a clustering of violent and risky sexual practices, suggesting important connections. This paper draws on Raewyn Connell's notion of hegemonic masculinity and reflections on emphasized femininities to argue that these sexual, and male violent, practices are rooted in and flow from cultural ideals of gender identities. The latter enables us to understand why men and women behave as they do, and the emotional and material context within which sexual behaviours are enacted.

In South Africa, while gender identities show diversity, the dominant ideal of black African manhood emphasizes toughness, strength and expression of prodigious sexual success. It is a masculinity women desire; yet it is sexually risky and a barrier to men engaging with HIV treatment. Hegemonically masculine men are expected to be in control of women, and violence may be used to establish this control. Instead of resisting this, the dominant ideal of femininity embraces compliance and tolerance of violent and hurtful behaviour, including infidelity.

The women partners of hegemonically masculine men are at risk of HIV because they lack control of the circumstances of sex during particularly risky encounters. They often present their acquiescence to their partners' behaviour as a trade off made to secure social or material rewards, for this ideal of femininity is upheld, not by violence per se, by a cultural system of sanctions and rewards. Thus, men and women who adopt these gender identities are following ideals with deep roots in social and cultural processes, and thus, they are models of behaviour that may be hard for individuals to critique and in which to exercise choice. Women who are materially and emotionally vulnerable are least able to risk experiencing sanctions or foregoing these rewards and thus are most vulnerable to their men folk.

We argue that the goals of HIV prevention and optimizing of care can best be achieved through change in gender identities, rather than through a focus on individual sexual behaviours.

## Introduction

### Intersections of HIV, gender power inequity in relationships and violence: evidence from epidemiology

In countries of sub-Saharan Africa with a predominantly heterosexual HIV epidemic, the prevalence in women climbs steeply in the late teens, which is five years before this occurs in men. Overall, a much greater proportion of the adult female population become infected [1,2]. Understanding this difference between women and men is critical for HIV prevention. While there are sex differences in susceptibility to HIV, which, like all sex differences, are rooted in biology [3], the patterns of prevalence have more complex origins. It is not biology, but gender differences in sexual socialization that are more important in influencing who women and men partner, when and in which circumstances.

Key here are differences in the way in which men and women position themselves and act as social beings, i.e., differences in socially defined and constructed ways of being a man or woman, and the power and possibilities so entailed. For it is gender, not sex, that is more influential in determining behaviour. In a given relationship, for example, a man may expect to lead and control sexual relations and his woman partner to comply, and he may feel entitled to have sex with other women, but expect her to remain faithful. Gender differences take many different forms in different settings, but an area of commonality lies in diffentials in power. There is strong evidence that gender power inequity in relationships, which is a cause of intimate partner violence, places women at enhanced risk of HIV infection.

South Africa is a country which exemplifies the dual epidemics of HIV and gender-based violence. It presently has 5.5 million people living with HIV, out of a population of about 47 million [1], the largest HIV epidemic in the world. The society is strongly patriarchal, and violence against women is widespread. South Africa's rate of rape has been found to be the highest of any INTERPOL member country [4], with more than 55,000 rapes reported to the police annually [5].

Intimate partner violence is also common. Research has found that between 25% and 55% of women have experienced physical intimate partner violence [6-8], and the rate of female homicide by an intimate partner is six times the global average [9]. In interviews, 42% of men disclose perpetration of intimate partner violence [10,11] and 28% disclose rape of a woman or girl [10].

These two epidemics have provided an important impetus for research into the nexus of gender and HIV, and the country provides an important opportunity to understand these problems and the implications of them for responses to HIV.

A decade of cross-sectional research from African countries, including Rwanda, Tanzania, South Africa and more recently, India, has consistently found women who have experienced partner violence to be more likely to be infected with HIV [12-15]. Two studies have shown that women who have been sexually coerced by male partners in Rwanda (n = 914) and Tanzania (n = 245) had a higher prevalence of HIV, with an adjusted odds ratio (aOR) of 1.89 (1.20, 2.96) in Rwanda, and 2.39 (1.21, 4.73) in Tanzania [12,13]. The Tanzanian study was conducted among women in a clinic offering voluntary testing and counselling, and it also showed that those aged under 30 who had ever experienced physical intimate partner violence were significantly more likely to have HIV [13].

In South Africa, among women (n = 1366) in antenatal care having HIV testing as part of treatment for prevention of mother to child transmission, those experiencing the greatest gender power inequity in relationships when compared with the most power equitable of three categories [aOR 1.56 (1.15, 2.11)], as well as those experiencing physical or sexual violence [aOR 1.53 (1.10, 2.04)] were more likely to be HIV seropositive [14]. Emerging evidence from yet unpublished longitudinal data from South Africa shows that women who have experienced intimate partner violence and have greater gender power inequity in relationships are at elevated risk of acquiring HIV. In both cases, there is a dose response relationship [16].

Research from India, analyzing data from husband-wife dyads (n = 20,425) that provided both intimate partner violence (IPV) exposure and HIV sero status has shown that abused wives face increased HIV risk, based both on the greater likelihood of HIV infection among abusive husbands and elevated HIV transmission within abusive relationships. This suggests that IPV functions both as a risk marker and as a risk factor for HIV among women [15].

In an effort to explain why partner violence and relationship gender power inequity should place women at risk of HIV, research has been conducted with men. This has shown that like their Indian counterparts, South African men who have been physically violent towards partners are more likely to be infected with HIV [10,15]. Some indications of why men who have been violent are more likely to be HIV infected can be seen in analyses that focus on the inter-relationship of gender-based violence perpetration and a range of risky sexual practices.

South African research shows an apparent clustering of violent, anti-social and risky sexual practices, suggesting that these are connected. Thus, men who have been violent towards intimate partners are more likely to rape, have large numbers of partners, drink heavily, not use condoms, have sex with prostitutes and engage in transactional sex [17]. Men who rape are more likely to have had transactional sex, be physically violent to partners, have large numbers of partners, drink heavily and engage in transactional sex [18]. Men who engage in transactional sex are more likely to be physically violent to partners, have large number of partners, drink heavily and rape [19]. In essence, men who are violent are more likely to be sexually risky, and vice versa. A key question is: what is the basis and nature of this connection? And what are the implications of these for HIV risk, prevention and care?

This paper seeks to move beyond the epidemiology and the measurement of behaviours and associations and enable us to understand these empirical findings. In so doing, we draw on theoretical resources from the area of critical men's studies, and in particular, the notion of hegemonic masculinity, initially developed and expounded by Raewyn Connell [20,21], and related discussion of femininities. We will describe the theoretical framework, discuss its relevance in terms of findings of South African research on hegemonic masculinity and femininities and their relation to HIV risk, and relate it to broader concerns in HIV prevention and care.

### Theoretical perspectives on men and gender

Feminist studies of sex and gender have historically foregrounded the oppression of women. Debates about the causes of, particularly, sexual oppression have frequently invoked a nature/nurture binary to explain global patterns of men's dominance over women. The former approach, which focuses on the genetic or physical to explain gender inequalities, has the major disadvantages of failing to explain diversity among men and among women and of lacking a model of how to make things better. Models that focus on how gender is a learned behaviour make more allowance for diversity and provide conceptual clarity about the forms that inequality take and how inequalities occur. Such analysis also can suggest gender equity interventions.

The focus on the social construction of gender has in the past quarter of a century generated a sophisticated literature on the gender identities of men and women, masculinities and femininities. This has permitted the conceptual inclusion of men within the ambit of gender studies, an initiative strongly associated with the theoretical work of Raewyn Connell [20,21].

Connell [20,21] describes the existence of multiple configurations of masculinity that are hierarchically organized and structured along lines of gendered domination (of men over women, of powerful men over less powerful men, of adult men over younger men). She identifies one masculine position that is dominant and refers to this as "hegemonic masculinity". It is this position that is generally associated with the subordination and oppression of women.

The concept of hegemony, drawn from Antonio Gramsci's work, refers to the exercise of power by creating consent through the establishment of accepted ideas or values. The concept is generally used descriptively to identify that form of masculinity that legitimates the subordination of women. It is in this sense that the framework enables an analysis of gender power while also allowing for the existence of divergent forms of male expression that may, for example, challenge the unquestioned right of men to this power.

There have been several interpretations of hegemonic masculinity by Connell herself [22] and others. Some focus on the fluidity and contested nature of the concept, while others stress the organizing, structured and structuring nature of hegemony. In this latter sense, hegemonic masculinity represents the dominant cultural model of idealized manhood. It is a frame used by individual men to judge their "success" as men. In a highly gender-inequitable country like South Africa, hegemonic masculinity mobilizes and legitimates the subordination and control of women by men. Conceived in this way, hegemonic masculinity is a necessary and integral element of patriarchy, the social organization that allocates, distributes and secures the power of men over women.

Hegemonic masculinity is characterized by a set of practices that both expresses men's power within the social system and serves to bolster this power. In essence, the practices flow from the hegemonic ideal. Implicit in the idea of "hegemony" is recognition that social ascendancy of this ideal of masculinity is not achieved through brute force, although violence may be used by men to bolster this ideal, but through a complex web of processes that extend into the organization of private life and cultural arrangements [21]. Thus, tenets of culture and religion and, for example, the operation of the legal system, may work to preserve the ascendancy of a particular cultural ideal of manhood.

Connell [21] argues that there is no equivalent notion of "hegemonic femininity" because there is more diversity in feminine ideals, although women are globally subordinated to men. She describes a form, or forms, of "emphasized femininity" that is characterized by compliance with women's subordination and an orientation towards accommodating the interests and desires of men. In other words, women "agree" with the unequal structuring of relations, do not challenge these relations, and ultimately collude in the unequal distribution of gender power with men. Other forms of femininity are shaped around strategies of resistance, and some combine compliance, resistance and cooperation [21].

Just as hegemonic masculinity is given power as a "cultural norm", forms of femininity that either in whole or in part emphasize compliance with this are expressed as cultural ideals of femininity, and are usually in some way socially rewarded. Women who adopt femininities based on resistance, or indeed engage in acts of resistance, can be marginalized and stigmatized. Patriarchal societies are heteronormative, that is, they require men and women to demonstrate their gender by actively participating in heterosex or affirming heterosexual desire [23].

While there are societally different ways in which this might be done, transgressions of heteronormativity are punished, and in South Africa, often violently so. The gang rape of African lesbian women and other instances of homophobic violence are particularly horrifying examples of this [24,25]. Having said this, it is important to note that gender identities change over time and that under particular circumstances, may change rapidly, for example, when legal or material contexts change dramatically. In South Africa, there is evidence that gender identities are indeed changing, although for our purposes, the persistence of gender violence remains a worrying continuity that shapes and binds forms of femininity and masculinity.

While hegemonic masculinity, and emphasized femininity, encompass practices that extend far beyond the arena of domestic, sexual and otherwise intimate relations with women (and men), it is the expression of these practices in these domains that is particularly pertinent to consideration of the intersections of gender power inequity and intimate partner violence and HIV risk. A lens of gender identity provides a frame through which we can begin to understand **why **men and women behave in the way that they do. It provides a way of reflecting on the emotional and material context within which sexual behaviours are enacted, in particular, the broader struggles, aspirations, desires and needs that motivate men and women's behaviour. It follows that only when we understand this, will we be able to change sexual behaviours (and thereby reduce the risk of HIV infection).

### Shape of masculinities and femininities among black Africans in South Africa

The gender order in South Africa under colonialism and apartheid was strongly racialized [26]. Two major features are relevant here. The first is that racial integration occurred to a very limited extent and this ensured that black African and white South Africans lived largely separate lives, connecting in the work place under conditions of inequality (whites dominating professional and business positions, and black Africans overwhelmingly limited to positions as labourers or subsistence farmers). This arrangement allowed for quite distinct racialized gender arrangements to persist, with perhaps the most notable feature being the retention of traditional forms of (male-dominated) authority (for example, chiefs). The second important feature was the emergence of distinctive gendered ideals for black and white men and women.

The material inequalities and associated spatial demography (with black Africans prohibited for a long period from living in cities unless in the service of white-owned industry, and therefore confined to increasingly impoverished rural areas), which are a feature of South African life to this day, impacted on constructions of masculinity and femininity. Offering a broad brush stroke description of gender topography always runs risks, but for our purposes, we will venture some generalizations. We do so even as we acknowledge that the changes unleashed by national political developments (especially the assumption of power by the African National Congress in 1994) and global economic forces have effected significant alterations to the stark picture that we paint here.

Until 1994, white men and women had the vote, had ready access to economic power or, at least, stable employment, and to forms of social and public status [26]. This influenced the ideals to which both white men and women aspired. White men were heavily invested in material achievement, public position and embodiment that found particular expression in sporting achievement. White women, on the other hand, were less vested in professional autonomy, even though they benefitted from free schooling in well-resourced institutions. Their identities were primarily built around children and the home.

For black African men and women, the material challenges of life were dominant. Men were generally employed in menial, poorly paid positions, and many found only seasonal, insecure ways of securing a livelihood or spent much of their time without any form of paid work [26]. This has made it difficult for the majority of black African men to vest their masculinity in material or professional achievement, and has increased the likelihood of finding masculine affirmation in homosocial (sometimes criminal) settings and in their relations with black women. Black African women, generally without the means to be economically independent, have often been dependent on black African men and this, together with cultural practices of respect, has promoted obedience and passivity as hallmarks of African femininity. With South Africa's history of colonialism and apartheid, all gender identities are in some ways marked by violence. We return to this theme shortly.

Historical perspectives on sex in South Africa reveal two competing discourses on sexuality. In one, rooted in Christianity, sex is located in marriage for procreation. The other reflects traditional black African ideas that sex is a normal and healthy and an essential feature of life for all ages, and something about which there should be openness and communication [26]. This latter discourse normalizes sex play in childhood and presents sexual exploration as a natural activity, including during adolescence. Historically, pre-marital penetrative sex was prohibited, but it is now the norm and, indeed, half of all black women have had a child by the age of 21, mostly outside marriage [27]. Within the frame of sexual openness, African women are constructed as sexual beings and sex is seen not just as normal in relationships, but as essential for their success [27,28]. Furthermore, in the domain of healing, sex is seen as a process of cleaning, and is commonly advised by traditional healers (and nurses) for a range of maladies [29].

For our purposes, it is important to make some statements specifically about gender in South Africa since 1994, when the country formally entered a period of transition, dismantling apartheid's edifice and constructing a new legal and policy framework for a non-racial democracy. This period has seen greater public diversity and fluidity in gender identities. The most obvious indication of this is the emergence of a public gay movement in the wake of the constitutional protection afforded to sexual orientation in the Bill of Rights in the Constitution in 1996, although the gay movement per se long preceded this [30].

For women, there has been a conspicuous emergence, primarily in urban settings, of "modern girl" femininities, associated with the exercise of independence, the use of specific fashion commodities and "explicit eroticism" [31]. This is an ideal of womanhood that is chiefly the domain of those women with access to (at least some) material resources. Whether these girls and young women seek political emancipation, or economic or sexual independence, the emergence of this phenomenon has drawn attention to the question of feminine agency.

Despite this diversity, there are clear patterns of power and dominance. While there is not one, single, dominant masculine form that serves as a model for all men, it is empirically clear that various racialized forms of masculinity are dominant. It is these masculinities that prescribe particular ways of being a man and legitimate gender-inequitable practices. One example of a black African hegemonic masculinity is found in the Zulu concept of *isoka*, an idealized heterosexual, virile man, who is desired by women, and whose prodigious sexual successes are the envy of other men [32]. Ethnographic research in the Eastern Cape province has shown that a key element of successful African manhood is heterosexual success and this is proved by being able to "win" desirable women, keep them (and thus prevent them from being seduced by others), and show evidence of being a man in control (of others) [33].

While the power of men is by no means established through the use of force, indeed the cultural foundations of patriarchy and processes through which it is maintained are broad and deep, and the use of violence, within limits and in particular contexts, is viewed by many, but not all, men as legitimate in pursuit of their goals [34]. This applies both in the public (for example, men resolving differences between one another using physical violence [35]) and private domains (where domestic violence, including femicide, is common).

South African masculinities all valorize the martial attributes of physical strength, courage, toughness and an acceptance of hierarchical authority, but most of all, they demand that men are able to exercise control (over women and other men) [36]. Within relationships with women, the expectations of establishing control provide space for the use of physical and sexual violence against women, in efforts both to achieve this and to demonstrate it. While men are not expected to injure women, and acts of extreme cruelty often incur familial and community wrath [34], the use of moderate violence by men (and in other circumstances, by women) is tolerated and generally is not viewed as evidence of weakness or lack of self-control.

With sex viewed as a need, particularly of men, but within context, also of women, wooing women with gifts, or exchanging money or other services for sex are seen as largely culturally acceptable practices [19]. Historically, sexual relationships between individuals were part of (subsumed) socially negotiated relationships between families, with marriages formalized through payment by men of *lobola*, the bridewealth. Nowadays, marriage occurs relatively late in adult life (at a mean age of 28 years for women [27]), if at all, and sex mostly happens outside marriage, and "serious" intent is demonstrated by gift giving. In this cultural milieu, it is easy for men to assume some form of patriarchal ownership over women and to establish or demonstrate this with physical violence. In this way, hegemonic masculinity inextricably links having multiple sexual partners with the subordination of women to male control, if necessary with the use of violence.

Other practices which flow from hegemonic masculinity involve sexual and other forms of risk taking. These include driving cars fast and dangerously, and heavy alcohol consumption; indeed, social norms around alcohol drinking are such that South Africa has the highest level of consumption per drinker of any country in the world [26,37]. Derision is cast on those who "fail" in navigating these risks without losing control or showing weakness, whether shown by their lives being destroyed by alcoholism or by becoming infected with HIV. Thus, blame is framed in terms of individual weakness, rather than being placed on the overarching gender order that provided the context within which these practices were and are encouraged [38,39].

In this way, hegemonic masculinity can be seen as a cultural ideal that links risky sexual practices and the use of violence and other controlling behaviours against women, particularly women partners. It is masculine-gendered identities, and the processes through which they are constructed, enacted and reproduced, that explain the clustering of violence and risky sexual practices seen in the epidemiological studies (discussed above). Viewed through this lens, these practices are seen as having meaning that extends well beyond the motives and rewards of the individual act.

With young black African women in the forefront of the HIV epidemic in South Africa, it is appropriate that we apply ourselves in the same way to young black African femininities. Our understanding of women's sexuality can be considerably advanced by reflecting in a similar manner on gender identity and the entailed meaning of practices. Emerging, yet unpublished research by the authors, based on extended qualitative interviews and participant observation over 10 months with women from the Eastern Cape, shows that the dominant idea of successful young womanhood is one where success is proven through being desirable to men. This is clearly complicit with hegemonic masculinity as it is framed in a way that encourages resonance, rather than discordance, with those ideas.

With worth of women assessed by men, women who wish to be "successful" are under massive pressure to conform to the dominant social order, including accepting the control by men. But there are other powerful forces at play. In a resource-poor setting, flirting and meeting with boyfriends provides hours of affordable entertainment. Thus, women have fun, compete and measure their desirability through flirting and encouraging proposals from men, while remembering that this is ultimately "proven" through having a boyfriend. Given the threat of being single to social status and self-esteem, and the risk of boredom, many women prefer to have more than one boyfriend (referred to as "walking on two legs") lest they split with one of them. The terms in Sotho and isiXhosa of *nyatsi *and *khwapheni *refer to secret concurrent partners, which is culturally accepted for women, as well as men, providing relationships are conducted in a manner respectful of the main partner, i.e., covertly [14,40].

With sex viewed as "natural", women's sexual desire is acknowledged, as is an expectation that sex should be pleasurable, preferably "flesh-to-flesh" sex and thus with no condom use [41]. While there has been a suggestion in literature on sexuality that it is a male requirement, authors have also found that women often oppose condom use because of concerns about their sexual pleasure, as well as a lingering suspicion that their chances of keeping their partners in the competitive world of multiple concurrency, are greater with flesh-to-flesh sex [41]. The emphasis on the heterosexual prerogative of men in a context of great gender inequalities has often led to treating women as sexually passive, simply waiting for men to propose and then acquiescing [42]. In some contrast to this, having multiple partners is on one level an expression of resistance to dependence on, and even control by, one man; yet the cultural acceptability of the practice allows women to do so without perceiving themselves as engaging in resistance to the gender order as a whole.

While the dominant ideal of femininity is fundamentally subordinate, women do not all experience controlling behaviour by their male partners to the same extent. Archetypically controlling boyfriends, however, expect to know where their partners are at all times, stop them seeing other men, expect to find them at home when they call, and to have them willing to free themselves from whatever they are engaged in and be ready for sex on demand [33]. It is hardly surprising that women with violent and controlling partners have been shown both to have more frequent sex and to use condoms less often [8,43-45]. Women are expected to avoid behaving in a way that threatens men's sense of control, failing which they are expected to endure and accept their physical punishment [33].

For African women, excusing male behaviour is an integral part of dominant femininity and essential for keeping the right man. In a practical sense that entails tolerance of violence (if he is violent), tolerance of his other partners (or when this fails, direction of aggression against them, rather than him), and ensuring that sex with the right man is "the best" (i.e., no condoms). This is supported by cultural wisdom, such as the saying that "beating is a sign of love". This dominant form of femininity thus requires women to be strong, and able to accept and cope with the stresses life brings, including those caused by women's subordinate position in their relationships.

Acquiescent femininity and hegemonic masculinity are both cultural ideals and are upheld by a system of sanctions and rewards. Women who do not comply, or express resistance, suffer marginalization and stigmatization. For example, divorce is an ultimate act of non-compliance, and for women in African culture, is strongly stigmatized and happens infrequently. In 2007, more white South Africans divorced than Africans (9935 versus 9055), despite the fact that the former represent only 9% of the population, compared to the latter group's 80% [46]. The position of these women was recently described by one older Xhosa woman politician, when she said, "In our language [isiXhosa] we have *iintombi *(unmarried girls) and *iintombazana *(married women). We have no word for women who divorce, we do not know where to put them." [47]

This is not to say that there is no social space in South Africa for gender difference. There are many men from across the social spectrum who adopt masculinities that incorporate counter hegemonic practices, such as engagement in childcare and caring for sick and disabled relatives, or support for gender equality and opposition to against violence against women [48,49]. There are also many women who are single mothers and economically independent of men [27]. But equally, it is important to read these behaviours through a historical and cultural lens.

In South Africa, the gendered division of labour has constantly evolved and shifted. Women historically have engaged in domestic work and caring [50]. They have adopted gender positions as "wives" in single-sex institutional settings [51-53], and women have run households that are economically independent of men [54]. The long historical trajectory shows the dynamism and fluidity of gender relations, but it does not show that these women and men resist the fundamental gender order that subordinates women to men [48]. It is possible to occupy apparently dissident gender positions without mounting an outright challenge to the gender order or supporting an alternative, gender-equitable vision of society.

Compliance with the dominant acquiescent femininity is rewarded, not just by men, but by other women. Women with desirable partners are admired by their peers, and respected in families and communities. Just as hegemonically masculine men seek amenable female partners so that their relationships can be relatively harmonious, rather than characterized by strong resistance, successful women desire hegemonic men [55]. Viewed as "real men", their displays of hegemonic masculinity are interpreted by many women as sexually and socially desirable, and research by the authors, and others, shows that men who practice more gender-equitable masculinities are often marginalized by women.

## Discussion

It is important for this argument not to be read in a way that is either culturally deterministic or victim blaming. We argue that in pursuit of hegemonic masculinity, as well as the dominant emphasised femininity, men and women are following ideals that have deep cultural roots and thus, models of behaviour that may be hard for individuals to critique and exercise real choices around. Indeed, we invoke a notion of choice for women with considerable caution, given the huge constraints on the power of young, impoverished women in a patriarchal society that has a marked age hierarchy.

Nonetheless, there is considerable diversity in the actual practices of men, choices of partners by women, and degrees of complicity, cooperation and resistance. There are women from across the social spectrum who resist gender inequality, and there is a proud history of women's movements in South Africa and of role models of women who have asserted considerable power of different forms within communities [56,57]. When interpreting women's decision making around partners and responses to male violence and controlling practices, it is apparent that women differ in the degree to which they accept and excuse these. While in some cases, this is a product of social and financial circumstances that leave no options, the visibility of this in the dating relationships of girls who are supported financially in their families reveals that the picture is more complex.

Women who experience more marked gender inequity in relationships and violence are placed at risk of HIV because they lack control of the circumstances of sex during particularly risky encounters, but their exposure to such gender inequity and violence is often related to complicity with an ideal of hegemonic masculinity. When women are acquiescent and accept male control and violence, their behaviour is considered as a trade off made from an expectation of social (or financial) reward. The degree to which women feel able to risk loss (or non-acquisition) of these rewards differs according to other dimensions of their material and emotional vulnerability. Thus, the poorest and most marginalized women, and those who have been rendered vulnerable in other ways, such as by abuse in childhood, may be least able to take the risk of displaying signs of non-conformity and resistance and of bucking the patriarchal trend of passively subordinating themselves to men.

### What are the implications for prevention and care?

Thus far, we have argued that sexual practices are rooted in and *flow from *(although not always in a consistent and linear way) gender identities, and therefore we need to address our attention to changing the bigger picture, rather than the individual behaviours. In real terms, this means focusing attention on building more gender-equitable and caring masculinities, and less acquiescent femininities. In so doing, interventions are needed at policy, service and community levels, as well as individual levels [58]. This needs to include, for example, investment in education, change to the national legal and policy framework related to gender equity, policy support for women's economic empowerment and property and inheritance rights, and strengthening the school curriculum and institutional environment so that it can promote gender equity and protect girl learners from violence and harassment in schools.

Both policy changes and service strengthening are needed to effectively enforce legislation that protects women and girls from gender-based violence and enables effective care and legal redress and protection for survivors. There is a need for initiatives at all levels to promote men's involvement in the care economy, including in South Africa, promoting the involvement of men as fathers, both financially and socially, in the lives of their children.

Interventions at an individual level and those that address community norms around gender and HIV have been developed in many settings. Some of these are gender sensitive, in that they recognise the specific needs and realities of men based on the social construction of gender roles. The better ones are "gender transformative" in that they seek to transform gender roles and promote more gender equity and thus address themselves to changing how men come to view themselves, and thus behave, as men [59].

Examples are interventions that have focused on changing harmful gender norms away from attitudes and behaviours that negatively impact on women's health and HIV risk through initiatives such as the Better Life Options for Boys that was implemented across 11 Indian states in schools with more than 8000 boys [60]. There are also examples of major national mass media initiatives, such as the Sexto Sentido campaign in Nicaragua, the Brothers for Life campaign in South Africa that seeks to change societal norms around masculinity, and the White Ribbon campaigns (initiated in Canada) that have focused on raising awareness about and changing norms on gender-based violence in many countries.

Sexto Sentido has been very extensively evaluated and shown to be effective in building gender-equitable attitudes, communication about HIV and condom use [61]. Other examples include the Program H group education intervention and social marketing campaign, developed in Brazil, that focused on improving sexual health and reducing HIV risk through changing gender norms and reducing violence. Its evaluation showed impact on gender attitudes and the prevalence of self-reported sexually transmitted infections [62]. Evaluation suggests that gender-transformative interventions are more effective than those that merely acknowledge or mention gender norms and roles.

The small, but emerging, body of literature on evaluations of HIV prevention behavioural interventions in sub-Saharan Africa has shown these to be generally unsuccessful, especially when using biological markers of sexual risk [63,64]. An exception is Stepping Stones. This intervention, first developed by Alice Welbourn for Uganda and now used in more than 40 countries, seeks to be gender transformative. Stepping Stones involves a participatory approach that includes critical reflection to encourage safer sexual practices through building more gender-equitable relationships. Evaluation of its effectiveness in a randomized controlled trial showed that it was successful in achieving a reduction both in a biological indicator (HSV-2 infections) in men and women and in perpetration of intimate partner violence over two years of follow up [65]. In the first year, changes in other male sexual practices were observed. It is appropriate to speculate whether Stepping Stones' success was a product of its engagement with gender identities, most conspicuously seen in a qualitative evaluation of its impact on those of men [66].

Interestingly, Stepping Stones had impact on women's HSV-2 incident infections, but measured change in sexual practices was not observed [65]. It is hard to know whether the changes in HSV-2 were a product of change in behaviours not measured as secondary outcomes, but the intervention did not impact on the most HIV-risky women as it did not reduce their likelihood of new HIV infection.

Qualitative research showed that the intervention was generally empowering for women and seemed to empower women in their minor sexual relationships (with *khwapheni*, secret concurrent partners), but there was more limited evidence of empowerment with their main sexual partner [66]. The evidence suggests that within the prevailing gender order, women perceived themselves to be unable to influence their partners' behaviour; they perceived that had they asserted themselves, the price would have been relationship break down. Some women accepted this, but given that so many of their short-term, and long-term, aspirations and sense of value were embedded in that relationship - and there has often been uncertainty about whether the next partner would be different - this was, for most, a price that was too high to pay.

This highlights the value of interventions in resource-poor settings that combine a focus on gender equity and broader structural interventions, such as seen in the IMAGE study, which combined microfinance with a programme on gender-based violence and related community action [67].

Ideas of masculinity and femininity also impact on HIV testing and thus access to treatment in different ways. Ideals of hegemonic masculinity that are predicated on toughness and being in control give little room for men to acknowledge vulnerability by testing for HIV and using health services. Their reluctance to do this has been well described. In South Africa, the 2008 National AIDS Survey showed that 43% of men and 57% of women had ever tested for HIV, and 20% of men and 29% of women said they had done so in the previous year [2].

There is evidence from services in multiple settings, and even global regions, that men enter antiretroviral treatment at lower CD4 counts than women and have a higher mortality on treatment [68,69]. The dominant model of femininity, in these respects, benefits women as they are diagnosed with HIV earlier and more likely to get into and do well on treatment. Changing constructions of masculinity are essential for encouraging men to engage with productive health seeking in an era of HIV.

Discussion of gender and HIV should not be concluded without reflecting on how HIV creates possibilities for gender transformation. The imperative for building safer sexual practices provides the possibilities of engagement with change in the gender order and encouraging more gender-equitable men [70]. Research also suggests that for men, the experience of having HIV can be part of the process of gender transformation [71]. For many men, being diagnosed with HIV is a life-changing event that shifts the way in which they position themselves with respect to their families and partners. Thus, faced with their own vulnerabilities, there are multiple accounts of men who engage in caring and support for their partners and extended families [48]. Similarly in his accounts of change to the Zulu ideal of *isoka *(the desirable heterosexual man, personified by men who had multiple sexual partners), Mark Hunter described how some men have come to realise that their very survival is predicted on their engagement with new ways of being men [72].

## Conclusions

There is a growing body of evidence showing that women who have experienced more gender power inequity in their relationship and gender violence are at greater risk of HIV. Since men who have been violent are more likely to be infected, it seems that women are least able to protect themselves when in relationships with men who pose the greatest risk for them.

Reflecting on the clustering of male violent and risky sexual practices, we have argued that these flow from dominant ideals of masculinity. Women's exposure to these is related to their adoption of femininities that forgive and accommodate male gender-inequitable and anti-social behaviour. These ideals of femininities are embedded in cultural processes that reward compliance. Women who are most vulnerable materially and emotionally are least able to reject them, and thus, most vulnerable to male violence and control, and consequently HIV.

Understanding sexual practices as flowing from gender identities helps us to understand why they are so hard to change, as well as how change should be approached. Evidence is suggesting that it is the underlying gender identities that must be changed to advance AIDS prevention and care.

Our understanding of how to change gender identities and build the gender equity to prevent HIV infections is still in its infancy; yet the experience of many countries teaches us that it is possible to move towards gender equity. Aligning the agendas of HIV prevention and building gender equity will help to extend human rights globally, as well as make HIV prevention more effective. However, resources for this work remain severely and disproportionately limited. It is essential that funders and politicians, researchers and activists work to ensure resources are available for the developing and testing of strategies to build more gender-equitable masculinities and femininities and to implement effective strategies to address the inseparably entwined problems of gender inequality, violence and HIV.

## Competing interests

The authors declare that they have no competing interests.

## Authors' contributions

This paper was written by both authors.
